# Impaired Neovascularization and Reduced Capillary Supply in the Malignant vs. Non-malignant Course of Experimental Renovascular Hypertension

**DOI:** 10.3389/fphys.2016.00370

**Published:** 2016-08-30

**Authors:** Andrea Hartner, Lisa Jagusch, Nada Cordasic, Kerstin Amann, Roland Veelken, Johannes Jacobi, Karl F. Hilgers

**Affiliations:** ^1^Department of Pediatrics and Adolescent Medicine, University Hospital of ErlangenErlangen, Germany; ^2^Department of Nephrology and Hypertension, University Hospital of ErlangenErlangen, Germany; ^3^Department of Nephropathology, University Hospital of ErlangenErlangen, Germany

**Keywords:** angiogenesis, aldosterone, malignant hypertension, neovascularization, PAI-1, VEGF

## Abstract

Malignant hypertension develops in some cases of hypertension but not in others. We hypothesized that an impaired neovascularization and a reduced capillary supply characterizes the malignant course of experimental hypertension. Two-kidney, one-clip renovascular hypertension was induced in rats; controls (sham) were sham operated. To distinguish malignant hypertension from non-malignant hypertension, we considered two factors: weight loss, and the number of typical vascular lesions (onion skin lesions and fibrinoid necroses) per kidney section of the nonclipped kidney. Animals in the upper half for both criteria were defined as malignant hypertensives. After 5 weeks, mean arterial blood pressure was elevated to the same degree in malignant hypertension and non-malignant hypertension whereas plasma renin and aldosterone were significantly higher in malignant hypertensives. The expression of plasminogen activator inhibitor-1 was elevated (up to 14-fold) in non-malignant but significantly more increased (up to 36-fold) in malignant hypertensive rats, compared to sham. As a bioassay for neovascularization, the area of granulation tissue ingrowth in polyvinyl discs (implanted subcutaneously) was reduced in malignant hypertension compared to non-malignant hypertension and sham, while there was no difference between non-malignant hypertension and sham. The number of renal and left ventricular capillaries was significantly lower in malignant hypertension compared to non-malignant hypertension, as was the number of proliferating endothelial cells. We conclude that an impaired neovascularization and capillarization occurs in malignant renovascular hypertension but not in the non-malignant course of the disease despite comparable blood pressure levels. This might contribute to the unique vascular lesions and progressive target organ damage observed in malignant hypertension.

## Introduction

Malignant hypertension is a serious complication of high blood pressure characterized by progressive target organ damage (Lip et al., 1995; Shantsila et al., 2010). Characteristic morphological lesions of the smaller blood vessels with proliferative onion skin lesions in the renal vasculature, and thrombotic microangiopathy are typical features of this condition. Presumably due to the availability of a variety of antihypertensive drugs, the prognosis of malignant hypertension has improved considerably in recent decades (Gonzalez et al., 2010). Nevertheless, successful treatment of this condition remains a challenge (Shantsila et al., 2010), and some patients continue to suffer from progressive target organ damage (Gonzalez et al., 2010). Further, it remains unclear why some patients develop malignant hypertension while others do not. Data from animal models of hypertension point to a role for the renin-angiotensin-aldosterone system in the development of malignant hypertension (Möhring et al., 1976; Luft et al., 1999; Hartner et al., 2006). Chronic activation of the renin-angiotensin system as seen in these models is associated with activation of inflammatory mechanisms, like overexpression of chemokines, macrophage infiltration, or activation of the complement system (Hilgers et al., 2001; Shagdarsuren et al., 2005). These changes, however, can also occur in non-malignant hypertension.

Endothelial dysfunction was shown to occur in malignant hypertension (Shantsila et al., 2011). Blockade of endothelin receptors prevented the development of malignant hypertension in some models of hypertension (Li et al., 1996; Orth et al., 1998), but was only partially effective in other models (Muller et al., 2000) and had even no effect at all in one model (Whitworth et al., 1995; Orth et al., 1998). Another possible endothelial pathomechanism is a disruption of endothelial regeneration. In some forms of thrombotic microangiopathy (e.g., preeclampsia or sirolimus-induced thrombotic microangiopathy), which share several morphological features with malignant hypertension, the expression or function of endothelial growth factors, like vascular endothelial growth factor (VEGF), is impaired (Levine et al., 2004; Sartelet et al., 2005). Inhibition of angiogenesis with anti-VEGF antibodies or inhibition of the VEGF receptor in cancer patients can induce hypertension or lead to an increase in preexisting hypertension (Sandler et al., 2004; Holden et al., 2005). There are even reports describing the development of malignant hypertension in patients treated with VEGF inhibitors (Caro et al., 2013). Moreover, an inhibition of VEGF receptor enhanced blood pressure in a rat model of salt-induced hypertension (Gu et al., 2009) and caused hypertension in mice (Facemire et al., 2009). Additionally, anti-angiogenic treatment in a transgenic rat model of hypertension caused glomerular damage in the kidneys with transformation to a malignant hypertensive phenotype (Advani et al., 2007). The similarity of the effects of anti-angiogenic therapy on the one hand, and spontaneous malignant hypertension on the other hand, prompted us to investigate the possibility that an impairment of angiogenic mechanisms may underlie the spontaneous development of malignant hypertension.

In our study we therefore used a rat model of malignant hypertension to compare the regenerative capacity of the vasculature in animals with malignant vs. non-malignant hypertension. We hypothesized that animals with malignant hypertension would exhibit a decreased neovascularization (measured by the disc angiogenesis bioassay) and a markedly impaired capillary supply of the kidney and heart compared to animals with non-malignant hypertension.

## Materials and methods

### Renovascular hypertension

Rats were housed in a room maintained at 22 ± 2°C, exposed to a 12 h dark/light cycle. The animals were allowed unlimited access to chow (#1320, Altromin, Lage, Germany) and tap water. All procedures performed on animals were done in compliance with the DIRECTIVE 2010/63/EU of the European Parliament and were approved by the local government authorities (Regierung of Mittelfranken, AZ 54-2532.1-51/12). Two-kidney, one-clip renovascular hypertension (2K1C) was induced in male Sprague-Dawley rats (Charles River, Sulzfeld, Germany) weighing 150–170 g by placing a silver clip of 0.2 mm internal diameter around the left renal artery through a flank incision under isoflurane anesthesia (using a vaporizer; starting with 5%, then followed by 1.5–2% via face mask) as previously described (Mai et al., 1995) (*n* = 59). Control animals underwent sham operation without placement of the clip (*n* = 25). Analgesia with subcutaneous buprenorphine injections (0.05 mg/kg) was provided post-operatively in all animals, and as needed later on. Weight and systolic blood pressure (tail cuff plethysmography) were measured weekly. During 5 weeks of follow-up, 12 2K1C rats died or had to be euthanized after severe weight loss and seizures and/or hemiplegia; these rats were not included in the analysis. Five weeks after clipping of the renal artery, the experiment was terminated and renal tissue was studied for the presence of onion skin lesions and fibrinoid necroses (assessed by two different blinded observers in two non-serial kidney sections) in all contralateral kidneys exposed to high blood pressure (Möhring et al., 1976; Hilgers et al., 2001). The criteria used for the definition of malignant hypertension in this study (see Section Results) were adapted from long-standing observations of weight loss and characteristic vascular lesions (Möhring et al., 1976; Hilgers et al., 2001) as hallmarks of the disease.

### Disc angiogenesis assay

After mounting of nitrocellulose filters on the polyvinyl alcohol sponges (Rippey, El Dorado Hills, CA, USA), the discs were autoclaved and implanted subcutaneously for 3 weeks as described elsewhere (Fajardo et al., 1988; Jacobi et al., 2005). Two discs per rat were implanted 2 weeks after clipping. Vascularization of the discs was assessed as described by Fajardo et al. (1988) by measuring the ingrowth of fibrovascular tissue (granulation tissue) which mirrors the ingrowth of vessels, as delineated in a more extensive method description published by Kowalski et al. (1992) and Jang et al. (2000). Fibrovascular tissue ingrowth was evaluated by two methods: photographs of the polyvinyl discs immediately after explantation and removal of the nitrocellulose filters were scanned for later quantification (see **Figures 4A–C**); and quantification of the tissue area observed in PAS-stained sections (after fixation of the disc with 3% paraformaldehyde, embedding in paraffin and cutting 2 μm sections) under the microscope in low-power views. The vascularized area was evaluated as percentage of the entire disc area. There was a good correlation between both methods (correlation coefficient [Spearman's rho]: 0.792); the data shown below are the results from the direct visual examination. The disc angiogenesis method was selected because it is a dynamic assay, because its development over time matches well with the length of the time period during which malignant hypertension is expected to occur, and because it is a rather small additional burden for the animals (compared to e.g., hindlimb ischemia).

### Blood pressure measurements

Systolic blood pressure was measured weekly by tail cuff plethysmography using a modification of previously described techniques (Williams et al., 1939) under light isoflurane anesthesia (starting with 2% for induction, followed by 0.5% via a face mask). At the end of the experiment rats were instrumented with femoral artery catheters for intraarterial blood pressure measurements as described previously (Menendez-Castro et al., 2011). Measurements were performed on the same day after termination of anesthesia and a recovery phase of 2 h in conscious animals via transducers connected to a polygraph (Hellige, Freiburg, Germany).

### Measurement of plasma creatinine, aldosterone, renin activity, VEGF, and soluble VEGF receptor

Blood for analysis was collected from indwelling catheters. Thereafter, rats were killed by bleeding in deep anesthesia. Plasma creatinine was analyzed using an automatic analyser Integra 800 (Roche Diagnostics, Mannheim, Germany). Plasma aldosterone was measured with a commercially available radioimmunoassay kit (Aldosterone Maia 12254, Serono Diagnostics, Freiburg, Germany). Plasma renin activity was measured with a commercially available radioimmunoassay kit (DiaSorin, Stillwater, MN, USA). Plasma VEGF and soluble VEGF receptor (Lip et al., 2000) were determined using commercially available ELISA kits (both R&D Systems, Wiesbaden, Germany) according to the manufacturer's protocol.

### Tissue sampling and histological analysis

After organ weighing, kidneys were decapsulated. Both poles of each kidney and the apical tip of the left ventricle was immediately snap frozen on liquid nitrogen for RNA extraction. One 6 mm slice of the kidney was snap frozen in TissueTek for immunofluorescence stainings while another 6 mm slice of the remaining kidney and left ventricular tissue was put in methyl-Carnoy solution (60% methanol, 30% chloroform, and 10% glacial acetic acid) for fixation. Paraffin embedded tissue was sectioned and stained with periodic acid Schiff's (PAS) reagent. Fibrinoid necroses and onion skin lesions were counted in PAS stained renal sections by two different blinded observers in two non-serial kidney sections in all contralateral kidneys exposed to high blood pressure.

### Immunohistochemistry

Tissue was processed as described (Menendez-Castro et al., 2013). Immunohistochemical detection of renin (rabbit antiserum kindly provided by Dr. Walter Fischli, Basel, Switzerland), collagen I (2150-1908, Biogenesis, Poole, England), α-smooth muscle actin 1A4 (Shantsila et al., 2010, DAKO, Hamburg, Germany), rat endothelial cell antigen (RECA, HIS 52, AbD Serotec, Düsseldorf, Germany), CD31 (TLD-3A12, BD Pharmingen, Heidelberg, Germany), and proliferating cell nuclear antigen [PCNA, PC10 (Shantsila et al., 2010), DAKO, Hamburg, Germany] was performed. All antibodies, except for CD31, were used on paraffin-embedded tissue sections. CD31 was used on cryosections. Interstitial collagen I was quantified in 30 medium-power views (magnification x200) by means of an 11 × 11 point grid. The percentage of grid points corresponding with a stained area was calculated. As a measure of kidney renin content the juxtaglomerular index was calculated. In each kidney, 100–200 glomeruli were counted, and the number of renin-positive glomeruli was expressed as a percentage of the total number of glomeruli counted. RECA or CD31 positive capillaries and PCNA positive cells were counted in 5 (heart) or 20 (kidney) medium power views (magnification x200 for RECA and PCNA on a Leitz microscope and magnification x240 for CD31 on a Nikon microscope). PCNA positive cells were also counted in 100 glomeruli per renal section. Proliferating endothelial cells were counted after double staining for RECA and PCNA in 6 medium power views of paraffin embedded tissue. Myocardial fibrosis was evaluated by looking for fibrotic areas (spanning at least 10,000 μm^2^ containing α-smooth muscle actin positive myofibroblasts) in α-smooth muscle actin stained left ventricular cross sections. Vascular damage in the left ventricle was evaluated by counting vascular cross sections with excessive thickening of the vascular wall and/or narrowing of the vascular lumen after staining for α-smooth muscle actin in a complete cross section of the left ventricle. To confirm the existence of blood vessels in discs, frozen discs were cut and stained with a RECA antibody detecting endothelial cells. All histological evaluations were done by a single investigator blinded to the group assignment.

### Western blot analysis

Frozen renal tissue was homogenized, protein samples were prepared as described (Menendez-Castro et al., 2014) and separated on a denaturing SDS-PAGE gel (Laemmli, 1970). After electrophoresis, the gels were electroblotted onto PVDF membranes (Hybond-P, GE Amersham, Munich, Germany), blocked with Rotiblock (Roth, Karlsruhe, Germany) for 1 h and incubated overnight with a primary antibody to VEGF-A (ab46154, Abcam, 1:1000). Protein bands were visualized with secondary horseradish peroxidase-conjugated IgG antibodies (Santa Cruz Biotechnology, 1:50000), using the ECL system (GE Amersham, Freiburg, Germany). Blots were quantified using a luminescent imager (LAS-1000, Fujifilm, Berlin, Germany) and an Aida 2.1 image analysis software (Raytest, Berlin, Germany). Loading of the blot was quantified by reprobing with an antibody to tubulin (Sigma, Taufkirchen, Germany, 1:10000).

### Real-time polymerase chain reaction (PCR) analyses

Myocardial and renal tissue was homogenized in RLT buffer reagent (Qiagen, Hilden, Germany) with an ultraturrax for 30 s, total RNA was extracted from homogenates by RNeasy Mini columns (Qiagen) according to the manufacturerer's protocol and real-time RT-PCR was performed (Gibson et al., 1996). First-strand cDNA was synthesized with TaqMan reverse transcription reagents (Applied Biosystems, Darmstadt, Germany) using random hexamers as primers. Reactions without Multiscribe reverse transcriptase were used as negative controls for genomic DNA contamination. PCR was performed with an ABI PRISM 7000 Sequence Detector System and TaqMan or SYBR Green Universal PCR Master Mix (Applied Biosystems), as described previously (Menendez-Castro et al., 2014). All samples were run in triplicates. Specific mRNA levels in hypertensive animals relative to sham operated controls were calculated and normalized to a housekeeping gene (18S) with the Δ-Δ-C_T_ method as specified by the manufacturer (http://www3.appliedbiosystems.com/cms/groups/mcb_support/documents/generaldocuments/cms_040980.pdf). Primer pairs used for experiments are shown in Table 1.

**Table 1:** **Primer pairs**.

	**Forward**	**Reverse**
VEGF-A	5′- AAC GAA AGC GCA AGA AAT CC -3′	5′- GCT CAC AGT GAA CGC TCC AG -3′
VEGF-B	5′- TGT ACC CAG GCC CCT GTG T -3′	5′- GCA CGT GCA TAA ACA TCT ATC CA -3′
VEGF-C	5′- CAG CAA GAC GTT GTT TGA AAT TAC A -3′	5′- GTG ATT GGC AAA ACT GAT TGT GA -3′
VEGF-D	5′- ACA CCG AGC AGT GAA GGA TG -3′	5′- AAC ACA GAC CGG GAT GAT CG -3′
VEGF-R1	5′- CGA CAC TCT TTT GGC TCC TTC TAA C -3′	5′- TGA CAG GTA GTC CGT CTT TAC TTC G -3′
VEGF-R2	5′- CCA CCC CAG AAA TGT ACC AAA C -3′	5′- AAA ACG CGG GTC TCT GGT T -3′
VEGF-R3	5′- CAT TGT GCA CGA AAA GCC CT -3′	5′- CGG GTA GCT TCA CCA TCT CG -3′
PlGF	5′- TAT GGA GCA CCA CTG ATG GA -3′	5′- TCA GCC AGG CTT TGA GAT TT -3′
PAI-1	5′- GTT CAC CAC TCC GGA TGG G -3′	5′- TGG TAG GGC AGT TCC AGG AT -3′
AP-1	5′- AAA CCA CAC GGC CAC CAT -3′	5′- TGG ATT TCA AGA CGG GAT GT -3′
	probe: TGG AGA TAG GAA CCA GCC TCT TGT CTC AGA CT	
AP-2	5′-GAC CAG TGG GCA TCG CTA CG -3′	5′- CAT TGT CCG AAT CCT TTG TGC T -3′
	probe: AAG GCA GCG AGG CAC ACT CTC TGT ATG AG	
Tie1	5′-GCC CCA GGA CAG CAT GAT TA-3′	5′- TCT CAC TGG GAT CCA CCA CA -3′
Tie2	5′-GCT GGA AGA ACG AAA GAC ATA CG -3′	5′- GCT CTC GTG CCA GTG AAG AGA -3′
18S	5′- TTG ATT AAG TCC CTG CCC TTT GT -3′	5′- CGA TCC GAG GGC CTC ACT A -3′

*List of primers pairs and probes used in the study*.

### Statistical analysis

One-way analysis of variance, followed by the Bonferroni *post-hoc* test, was performed to test significance of differences between groups. For repetitive measurements (time course of systolic blood pressure), two-way analysis of variance was used. A *p* < 0.05 was considered significant. To assess the dis/agreement between different observers or different methods to quantify continuous measurements, Spearman's correlation coefficients (Spearman's rho) were calculated. Calculations were carried out using the SPSS 19 software (IBM, Ehningen, Germany).

## Results

During 5 weeks of follow-up, 12 2K1C rats died or had to be euthanized after severe weight loss and seizures and/or hemiplegia. This substantial mortality is in line with the survival of animals in similar rat models and reflects the severity of the malignant form of the disease (Kantachuvesiri et al., 1999; Biala et al., 2010). In the following paragraphs, results from 3 groups (sham operation = sham, non-malignant hypertension = NMH and malignant hypertension = MH) are shown whereas results for the 19 hypertensive 2K1C rats which were classified as “undetermined” with regard to the presence of malignant hypertension (see next paragraph) are presented in the online supplement (Please see Table S1, Figures S2, S3)

### Criteria for malignant hypertension

**Figure 1:**
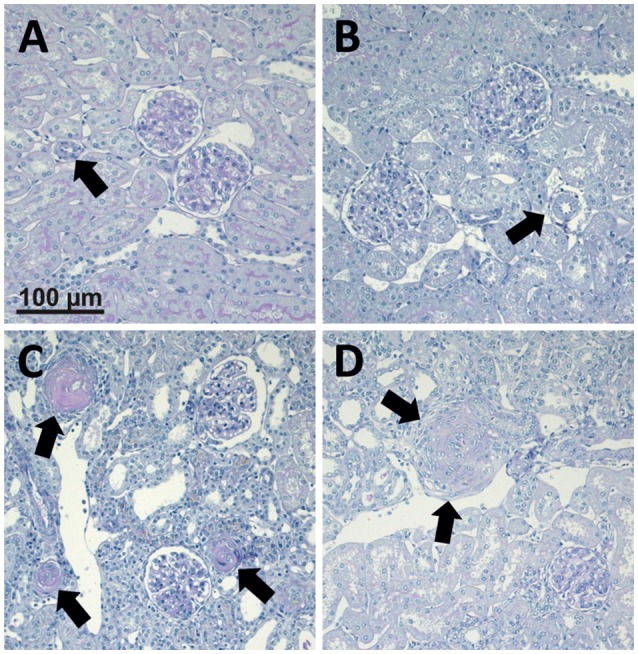
**Photomicrographs of PAS stained renal sections representative for 25 control animals, 15 non-malignant hypertensive animals, and 13 animals with malignant hypertension**. **(A)** Renal section from a sham OP control; **(B)** Renal section from an animal with non-malignant hypertension. Arrows mark renal artery. **(C,D)** Renal sections from animals with malignant hypertension (**C**, arrows mark fibrinoid necroses. **D**, arrows mark onion skin lesion).

The occurrence of malignant hypertension was defined as the presence of weight loss in these otherwise still growing rats and characteristic vascular lesions in the contralateral kidney (Figure 1) exposed to high blood pressure. Because malignant hypertension may develop in 2K1C rats at different time points, we suspected that some animals might present an intermediate course. Therefore, we performed split-half analyses for both criteria: weight loss and number of characteristic vascular lesions (onion skin lesions and fibrinoid necroses). To avoid the need for arbitrary definitions of “weight loss” (e.g., what percentage of weight had to be lost? For how many days? How to deal with several episodes of weight loss during observation?), the actual weight gain between the 2K1C procedure and the end of the study was used in the split-half analysis for “weight loss.” In these growing rats, the animals with the most weight loss had obviously the lowest weight gain during this time (see Figure S4). Regarding the number of vascular lesions, the correlation between both observers (Spearman's rho) was 0.93. Hypertension was considered malignant if a rat was in the upper 50% for both criteria (*n* = 13, MH), or non-malignant if animal was lower 50% for both criteria (*n* = 15, NMH). Rats in the upper 50% of one criterion but in the lower 50% for the other were considered as “undetermined.”

### End organ damage is more prominent in malignant hypertension

**Figure 2:**
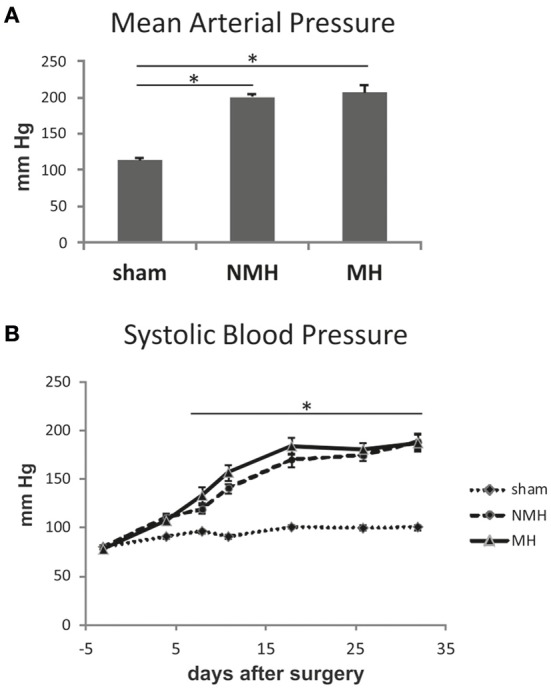
**Blood pressure measurements**. **(A)** Intraarterial blood pressure measurement in conscious animals at the end of the experiment. **(B)** Weekly non-invasive blood pressure measurements using tail cuff plethysmography under isoflurane anesthesia. Data are means ± sem. ^*^p < 0.05 vs. sham OP control. NMH, non-malignant hypertension; MH, malignant hypertension.

**Figure 3:**
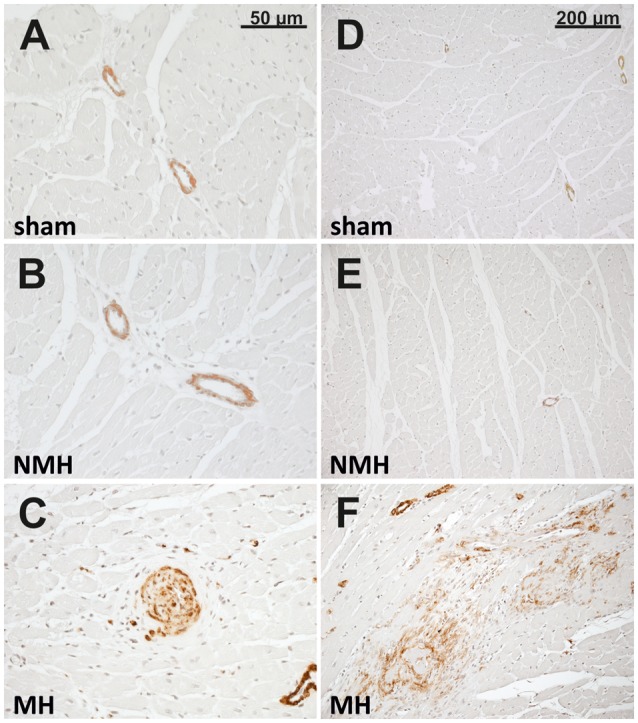
**Histology of the left ventricle**. Photomicrographs (representative for 24 control rats, 13 rats with non-malignant hypertension, and 12 rats with malignant hypertension) of left ventricular sections stained with α-smooth muscle actin in sham OP controls (**A,D**; sham), non-malignant hypertension (**B,E**; NMH) and malignant hypertension (**C,F**; MH) with **(C)** showing a vascular lesion and **(F)** showing a fibrotic myocardial area.

For sham, MH and NMH groups, body weights were comparable at the time of clipping of the renal artery (Table 2). Weight gain over the 5 week observation period was somewhat reduced in NMH compared to sham, but was most intensely reduced in MH as compared to sham or NMH (Table 2 and data in Figure S3). At the time of sacrifice the organ weights of the right unclipped kidneys of MH and NMH were increased to the same degree as compared to sham (Table 2). The left ventricular weights increased with hypertension, however, they were higher in MH compared to NMH (Table 2), despite comparable blood pressure increases in NMH and MH (Figure 2). In accordance with the results from the blood pressure measurements, the induction of renin expression in the juxtaglomerular apparatus after clipping of the left kidney was comparable in NMH and MH (Table 2). On the other hand, plasma renin activity was more prominently increased in MH compared to NMH (Table 2). Serum creatinine levels and collagen I deposition in the right kidney as markers of renal damage were both increased in MH only (Table 2). While tubulointerstitial cell proliferation (PCNA positive cells) was increased in both NMH and MH to a similar degree, glomerular and left ventricular cell proliferation was increased in NMH only (Table 2) (For representative photomicrographs of renin, collagen I and PCNA stained renal sections see data in Figure S5). Evaluation of vascular lesions in the left ventricle revealed a significantly higher incidence of vascular lesions in MH compared to NMH or sham (4.50 ± 0.72 vascular lesions per ventricular cross section in MH vs. 1.38 ± 0.35 in NMH and 0.17 ± 0.08 in sham, *p* < 0.05) as depicted in Figure 3. Fibrotic areas in left ventricular tissue were only detected in MH (Figure 3F).

**Table 2:** **Body and organ weights, physiologic parameters and markers of organ damage**.

	**Sham-OP control**	**Non-malignant hypertension**	**Malignant hypertension**
Body weight at OP (g)	151.2 ± 1.4	151.7 ± 1.8	151.8 ± 2.4
Body weight at sacrifice (g)	381.0 ± 8.0	343.6 ± 9.4^*^	251.4 ± 7.5^*^^,^^†^
Kidney weight (right, unclipped) (g)	1.22 ± 0.02	1.44 ± 0.06^*^	1.40 ± 0.10^*^
Kidney weight (left, clipped) (g)	1.20 ± 0.03	0.94 ± 0.07^*^	0.86 ± 0.08^*^
Left ventricular weight (g)	0.78 ± 0.01	0.93 ± 0.09	0.85 ± 0.08
Relative left ventricular weight (mg/g)	2.05 ± 0.04	2.69 ± 0.26^*^	3.42 ± 0.31^*^^,^^†^
Renin in clipped kidney (juxtaglomerular index, %)	22.4 ± 1.5	30.5 ± 1.8^*^	31.2 ± 3.0^*^
Serum creatinine (mg/dl)	0.20 ± 0.01	0.22 ± 0.01	0.47 ± 0.06^*^^,^^†^
Serum urea (mg/dl)	37.7 ± 1.1	45.0 ± 2.9	122.3 ± 18.3^*^^,^^†^
Plasma VEGF (pg/ml)	67.63 ± 6.30	73.63 ± 7.83	105.82 ± 15.67^*^
Plasma renin activity (ng Ang I/ml/h)	8.08 ± 1.78	17.83 ± 3.20^*^	41.61 ± 5.57^*^^,^^†^
Collagen I deposition in unclipped kidney (% area stained)	4.66 ± 0.44	4.98 ± 1.02	10.16 ± 1.53^*^^,^^†^
Tubulointerstitial PCNA-positive cells in unclipped kidney (no per cortical view)	4.43 ± 2.23	10.71 ± 1.85^*^	11.79 ± 1.60^*^
Glomerular PCNA-positive cells in unclipped kidney (no per glomerulus)	0.56 ± 0.05	1.09 ± 0.13^*^	0.49 ± 0.09^†^
PCNA-positive cells in left ventricle (number per view)	1.82 ± 0.23	3.53 ± 0.62^*^	1.33 ± 0.46^†^

*Data are means ± sem*.

* *p < 0.05 vs. sham*,

† *p < 0.05 vs. non-malignant hypertension*.

### Neovascularization of discs and capillarisation of target organs is reduced in malignant hypertension

**Figure 4:**
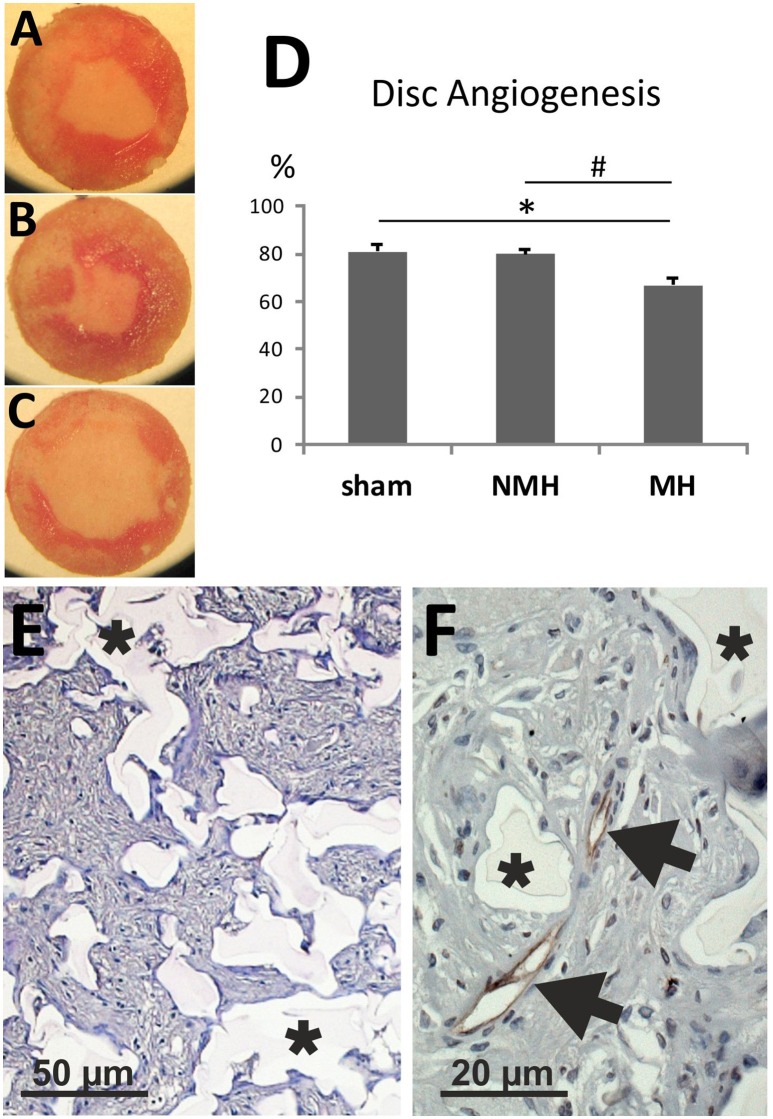
**Disc angiogenesis assay**. **(A)** Disc from a control animal (representative for *n* = 13). **(B)** Disc from an animal with non-malignant hypertension (representative for *n* = 9). **(C)** Disc from an animal with malignant hypertension (representative for *n* = 9). **(D)** Evaluation of the disc angiogenesis assay. ^*^p < 0.05 vs. sham OP control, ^#^*p* < 0.05 vs. non-malignant hypertension. **(E)** Exemplary photomicrograph of a PAS stained section of a disc from a control animal showing immigrated cells stained in purple (granulation tissue). Asterisks mark disc matrix. **(F)** Photomicrograph of aRECA stained section of a disc showing endothelium of blood vessels (arrows). Asterisks mark disc matrix.

**Figure 5:**
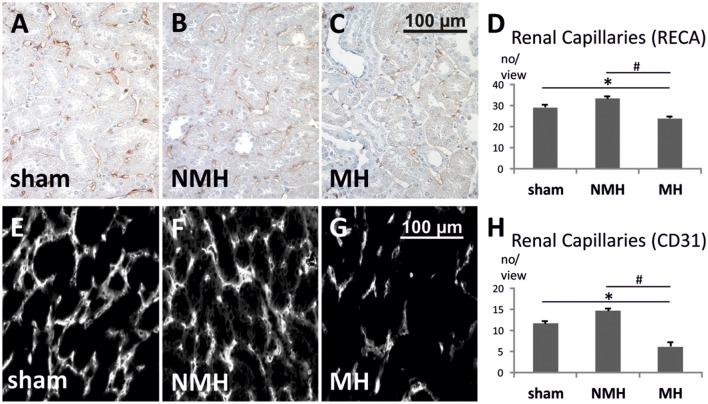
**Quantification of renal capillaries**. **(A–C)** Photomicrographs of RECA stained renal sections (representative for 25 controls, 14 non-malignant hypertensives, and 12 malignant hypertensive rats). **(D)** Evaluation of RECA stained capillary cross sections in paraffin-embedded renal tissue. **(E–G)** Photomicrographs of immunofluorescent CD31 stained renal cryosections (representative for 9 controls, 6 non-malignant hypertensives, and 9 malignant hypertensive rats). Evaluation of CD31 stained capillary cross sections in renal tissue. **(H)** Evaluation of CD31 stained capillary cross sections in frozen renal tissue. Please note that the absolute number of capillaries per view is not directly comparable between panels **(A–D)** on the one hand, and panels **(E–H)** on the other hand, due to differences in tissue fixation, sectioning, staining procedures, antibodies employed, detection methods (immunofluorescence vs. immunohistochemistry) and microscopes (see Section Materials and Methods for details). Data are means ± sem. ^*^p < 0.05 vs. sham OP control, ^#^*p* < 0.05 vs. non-malignant hypertension. NMH, non-malignant hypertension; MH, malignant hypertension.

**Figure 6:**
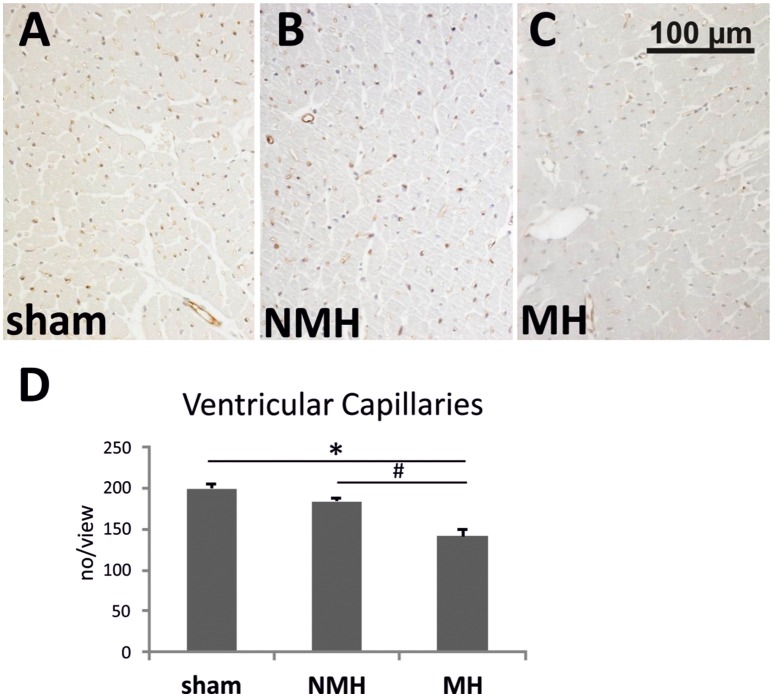
**Vascularisation of the left ventricle**. **(A–C)** Photomicrographs of RECA stained heart sections (representative for 15 controls, 9 non-malignant hypertensives, and 11 malignant hypertensive rats). **(D)** Evaluation of RECA stained capillary cross sections in heart tissue. Data are means ± sem. ^*^p < 0.05 vs. sham OP control, ^#^*p* < 0.05 vs. non-malignant hypertension. NMH, non-malignant hypertension; MH, malignant hypertension.

**Figure 7:**
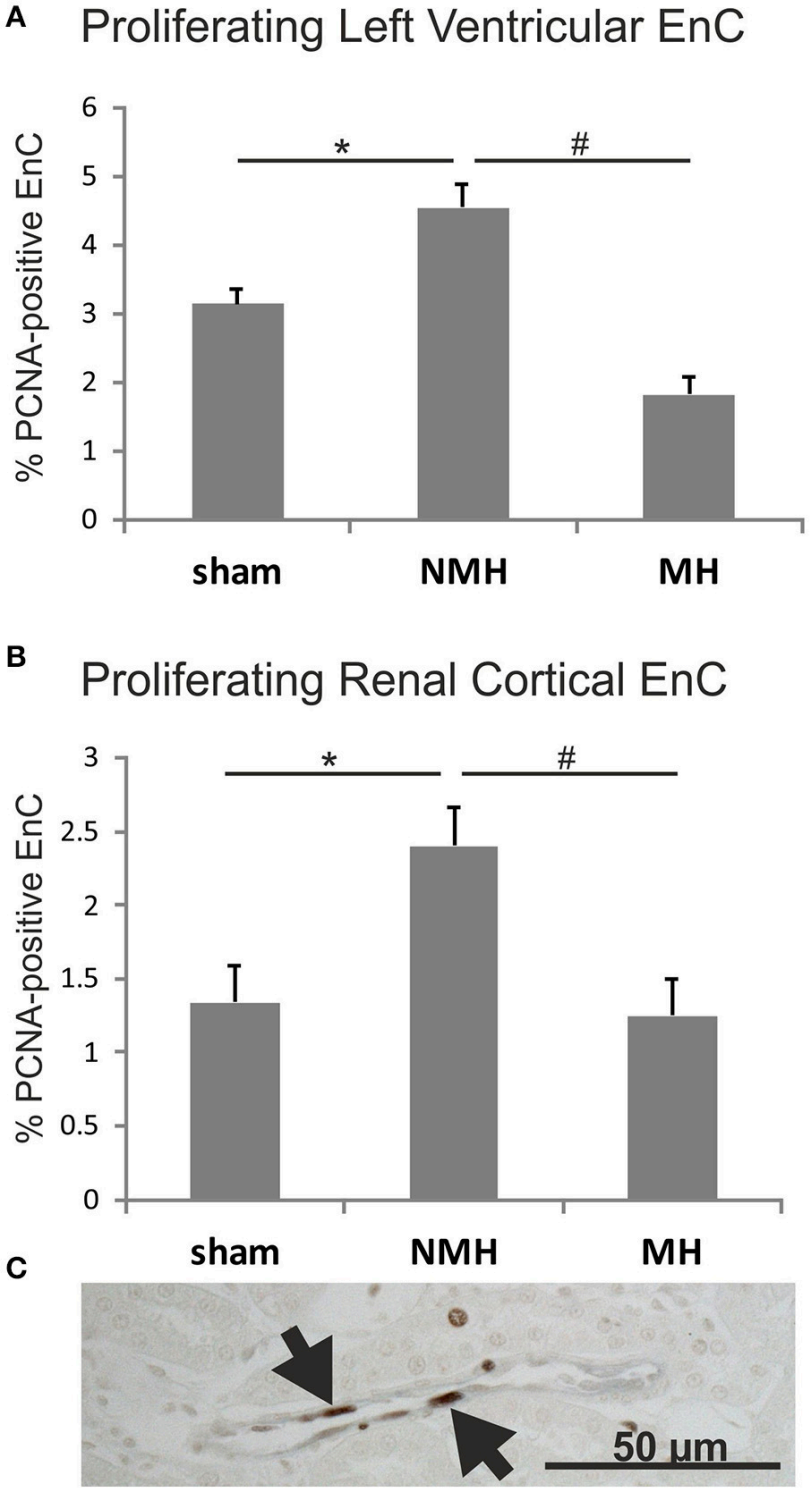
**Proliferation of endothelial cells (A), in the left ventricle and (B), in renal tissue**. Exemplary photomicrograph **(C)** showing double staining for the endothelial cell marker RECA (black) and the proliferation marker PCNA (brown, arrows) in renal tissue of a control animal. Data are means ± sem. ^*^p < 0.05 vs. sham OP control, ^#^*p* < 0.05 vs. non-malignant hypertension. EnC, endothelial cells; NMH, non-malignant hypertension; MH, malignant hypertension.

**Figure 8:**
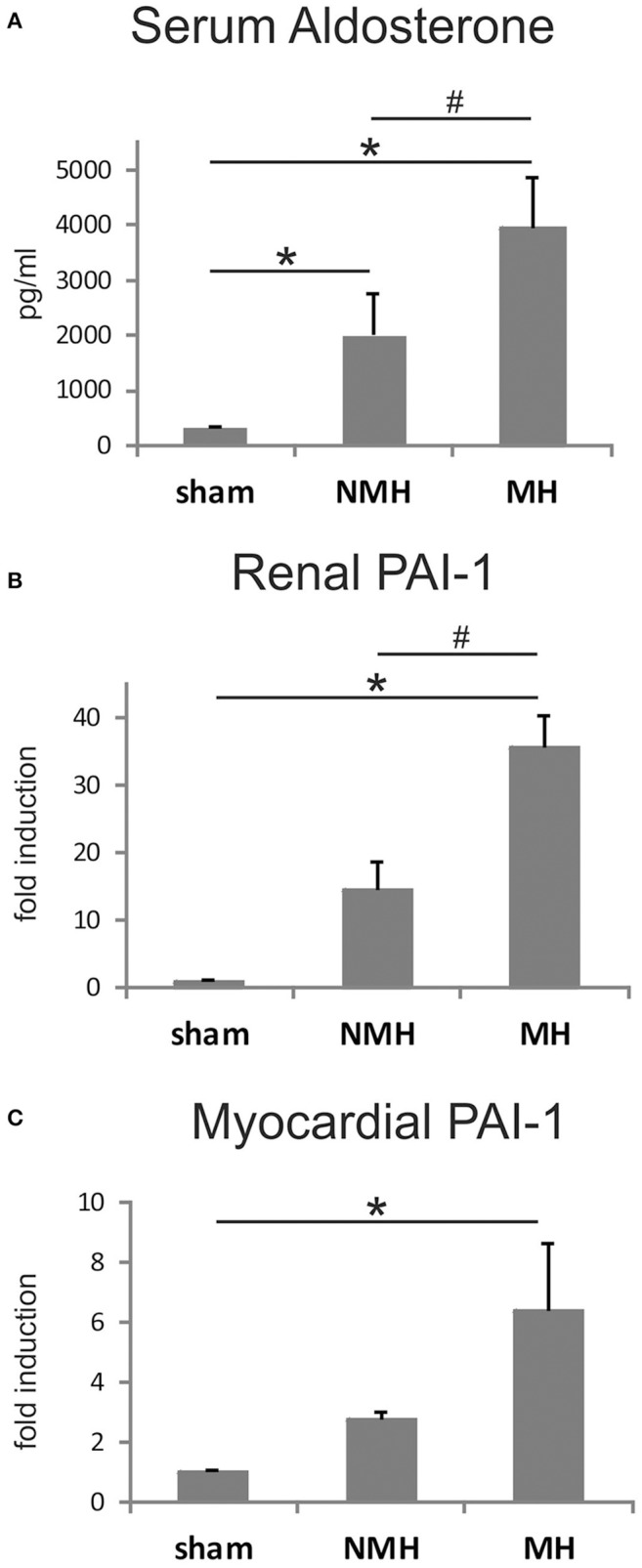
**Measurements of plasma aldosterone levels and tissue PAI-1 expression**. **(A)** Serum aldosterone levels, **(B)**, renal PAI-1 expression, and **(C)**, left ventricular PAI-1 expression. Data are means ± sem. ^*^p < 0.05 vs. sham OP control, ^#^*p* < 0.05 vs. non-malignant hypertension. NMH, non-malignant hypertension; MH, malignant hypertension.

To evaluate neovascularization, a disc angiogenesis assay was performed. There was a high agreement between both blinded observers regarding the quantification of the area of vascular ingrowth [correlation coefficient (Spearman's rho): 0.929]. The area of vascularisation was similar in sham and NMH, but significantly reduced in MH (Figure 4). Vascularisation of the right unclipped kidney was also reduced in MH compared to sham and NMH, as assessed by two different markers of capillaries (Figure 5). Similar results were obtained in heart tissue, where the capillarisation of the left ventricular myocardium was reduced in MH compared to sham and NMH (Figure 6). In both left ventricular and renal tissue of NMH, more proliferating endothelial cells were observed than in MH (Figures 7A–C). Serum aldosterone levels were higher in both NMH and MH compared to sham, but in MH serum aldosterone levels were significantly higher than in NMH (Figure 8A). The induction of renal and left ventricular plasminogen activator inhibitor-1 (PAI-1) was more prominent in MH than in NMH (Figures 8B,C).

### Expression of VEGF and VEGF receptors is not reduced in malignant hypertension

Plasma levels of VEGF were comparable between sham and NMH, while significantly increased in MH (Table 2). Soluble VEGF receptor 1 was not detectable in plasma in all but two animals (only one MH and one NMH rat exhibited levels slightly above detection limit, data not shown).

In the heart, the expression levels of VEGF A, B, C, D and VEGF receptors 1, 2, 3 as well as the angiogenic factor placental growth factor (PlGF) remained unchanged in NMH and MH, with the exception of VEGF A expression which was significantly reduced in MH compared to controls and VEGF D expression which was significantly increased (Table 3). The expression of the angiogenic factor angiopoietin-1 was reduced in NMH and MH to a similar degree, while the expression of angiopoietin-2 remained unchanged. Expression analysis of the angiopoietin receptors Tie1 and Tie2 revealed a slight reduction of Tie1 in MH and a slight increase of Tie2 in NMH.

**Table 3:** **Expression of angiogenic factors and receptors in the left ventricle [fold induction]**.

**Left ventricle**	**Sham-OP control**	**Non-malignant hypertension**	**Malignant hypertension**
VEGF-A	1.00 ± 0.09	0.76 ± 0.08	0.56 ± 0.06^*^
VEGF-B	1.00 ± 0.04	0.95 ± 0.11	0.72 ± 0.09
VEGF-C	1.00 ± 0.07	1.27 ± 0.18	1.12 ± 0.33
VEGF-D	1.00 ± 0.09	1.2 ± 0.10	1.95 ± 0.44^*^
VEGF-receptor 1	1.00 ± 0.05	0.99 ± 0.16	0.95 ± 0.14
VEGF-receptor 2	1.00 ± 0.09	1.06 ± 0.14	0.75 ± 0.10
VEGF-receptor 3	1.00 ± 0.08	1.33 ± 0.16	1.67 ± 0.60
PlGF	1.00 ± 0.04	1.42 ± 0.19	1.18 ± 0.21
Angiopoietin-1	1.00 ± 0.10	0.48 ± 0.04^*^	0.39 ± 0.07^*^
Angiopoietin-2	1.00 ± 0.10	1.31 ± 0.17	1.00 ± 0.11
Tie1	1.00 ± 0.05	1.38 ± 0.17	0.88 ± 0.13^†^
Tie2	1.00 ± 0.06	1.43 ± 0.16^*^	1.05 ± 0.09

*Data are means ± sem*.

* *p < 0.05 vs. sham*,

† *p < 0.05 vs. non-malignant hypertension*.

**Figure 9:**
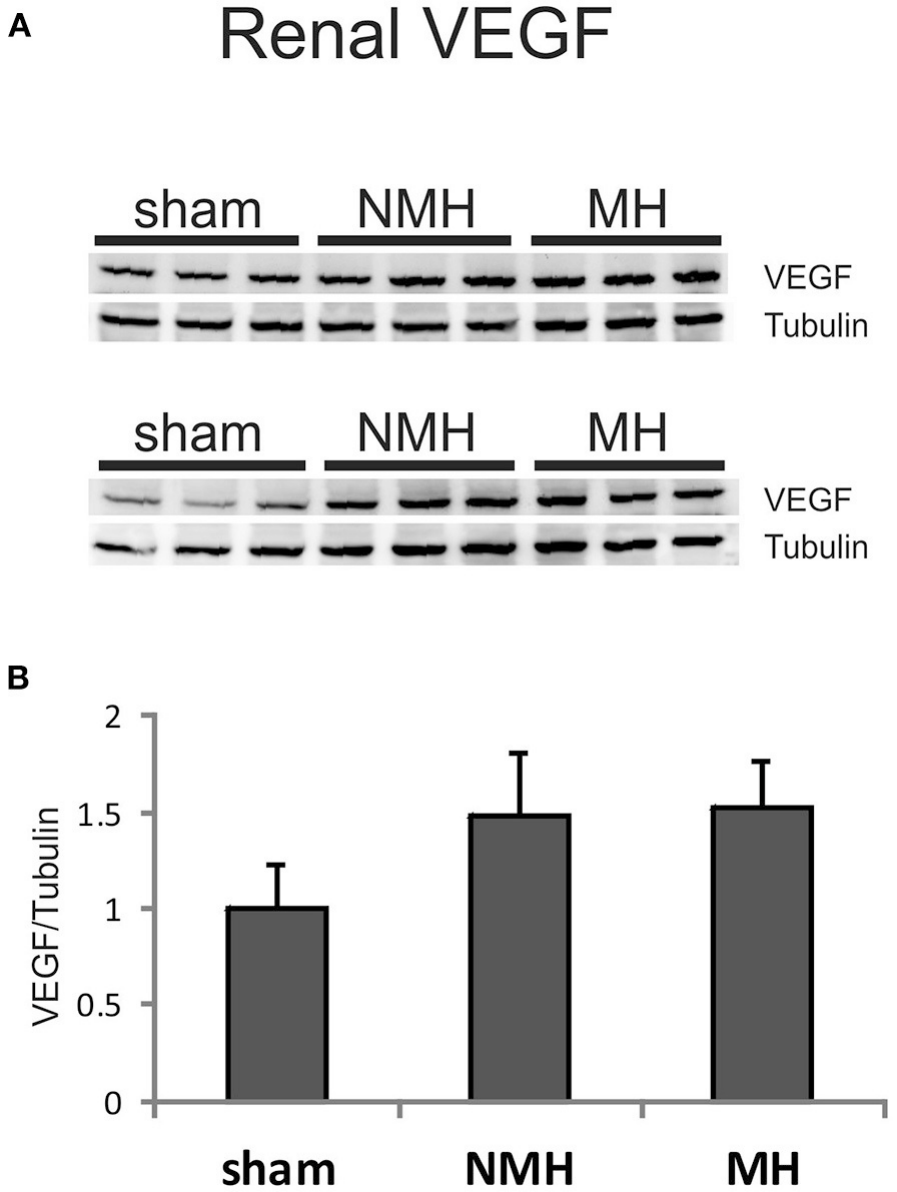
**Protein expression of VEGF-A in the right (non-clipped) kidney as assessed by Western blot analysis**. **(A)** Representative photographs of Western blots. **(B)** Densitometric evaluation of VEGF protein in relation to the housekeeper tubulin. Data are means ± sem. NMH, non-malignant hypertension; MH, malignant hypertension.

The expression of VEGF A, B, C, D, PIGF, VEGF receptors 1, 2, 3 and angiopoietin-2 was induced in the right kidneys of MH and NMH (Table 4). The induction of renal VEGF B, C, D and VEGF receptor 3 as well as PlGF and angiopoietin-2 expression was significantly higher in MH than in NMH (Table 4). Western blot evaluation of VEGF protein in the right kidneys did not yield significant changes but showed the same trend as observed by RT-PCR for gene expression of VEGF-A (Figure 9).

**Table 4:** **Expression of angiogenic factors and receptors in the right (non-clipped) kidney [fold induction]**.

**Right (non-clipped) kidney**	**Sham-OP control**	**Non-malignant hypertension**	**Malignant hypertension**
VEGF-A	1.00 ± 0.23	2.23 ± 0.20^*^	2.72 ± 0.26^*^
VEGF-B	1.00 ± 0.21	2.62 ± 0.33^*^	3.87 ± 0.24^*^^,^^†^
VEGF-C	1.00 ± 0.27	2.70 ± 0.34^*^	4.94 ± 0.40^*^^,^^†^
VEGF-D	1.00 ± 0.31	2.57 ± 0.34	4.44 ± 0.52^*^^,^^†^
VEGF-receptor 1	1.00 ± 0.21	2.31 ± 0.31^*^	3.07 ± 0.27^*^
VEGF-receptor 2	1.00 ± 0.23	2.33 ± 0.31^*^	2.38 ± 0.23^*^
VEGF-receptor 3	1.00 ± 0.29	2.92 ± 0.47^*^	5.77 ± 0.55^*^^,^^†^
PlGF	1.00 ± 0.19	2.73 ± 0.35	4.13 ± 0.24^*^^,^^†^
Angiopoietin-1	1.00 ± 0.87	1.59 ± 0.18	1.82 ± 0.14^*^
Angiopoietin-2	1.00 ± 0.23	1.84 ± 0.21^*^	3.08 ± 0.18^*^^,^^†^
Tie1	1.00 ± 0.25	1.43 ± 0.15	1.54 ± 0.10
Tie2	1.00 ± 0.25	1.62 ± 0.18	1.78 ± 0.13^*^

*Data are means ± sem*.

* *p < 0.05 vs. sham*,

† *p < 0.05 vs. non-malignant hypertension*.

## Discussion

In our animal model of malignant hypertension organ damage was associated with reduced capillarisation in the kidney and the heart. Such an observation could be due to a reduced formation of new capillaries, or to a loss of capillaries by e.g., fibrotic processes. On the one hand, the reduced number of proliferating cells in glomeruli and the left ventricle of animals with malignant hypertension points to the former mechanism. On the other hand, fibrotic processes in heart and kidney especially in malignant hypertensives could lead to a loss of capillaries. The reduced capillarisation could not be attributed to an altered expression of several angiogenic growth factors. To clarify this issue, we finally used a disc angiogenesis model as a direct *in vivo* bioassay of the formation of new vessels. Here, we observed reduced neovascularization only in malignant hypertension but not in the non-malignant course of the disease. However, we performed only an endpoint measurement of these processes, not an investigation at different time points, which is an important limitation of our study.

The relationship between hypertension and angiogenic factors and processes is often puzzling, and conflicting observations have been reported. On the one hand, capillary rarefaction and a reduced angiogenic potential was observed in some animal models of hypertension (Belabbas et al., 2008; You et al., 2008). Evidence for a reduced microvascular supply has been described in humans with established essential hypertension (Antonios et al., 1999a), with borderline essential hypertension (Antonios et al., 1999b) and even in young adults with a predisposition to hypertension (Noon et al., 1997). On the other hand, in prehypertensive spontaneously hypertensive rats, angiogenesis was found increased (Hudlett et al., 2005), and increased levels of angiogenic growth factors were measured in hypertensive patients with target organ damage (Nadar et al., 2005). In rats with renovascular hypertension glomerular VEGF A expression was induced (Advani et al., 2007). Plasma VEGF levels were clearly induced in patients with essential hypertension (Belgore et al., 2001). Our most striking observation was the reduced neovascularization and capillarization in animals with malignant hypertension, but not in animals with non-malignant hypertension, despite the identical procedure to induce hypertension, and the comparable blood pressure levels in both groups. The results of our study indicate that the potential to form and/or preserve new vessels is not uniform in hypertensive disease and might be dependent on additional factors which also determine the severity of hypertensive organ damage.

Our observations depend on the ability to distinguish between malignant and non-malignant hypertension which was only possible in retrospect from the animal's course during follow-up. Moreover, this distinction was rendered even more difficult by the fact that we cannot be sure that a given animal would not develop malignant hypertension later on if given the chance. We therefore used rather strict definitions of malignant and non-malignant hypertension, and excluded a substantial number of animals with an intermediate course. The spontaneous occurrence of the malignant hypertensive phenotype in some but not all 2K1C rats has long been recognized (Möhring et al., 1976), marked by weight loss, characteristic vascular lesions, and a more marked stimulation of renin and aldosterone. The relatively small (or even absent) difference of blood pressure between malignant and non-malignant hypertension, despite markedly higher plasma renin and aldosterone levels, is surprising but has been noted previously (Möhring et al., 1976). In fact, some animals experience a drop of blood pressure as malignant hypertension occurs (Möhring et al., 1976). This observation may be due to the loss of sodium and volume via the kidney (Möhring et al., 1976). In this situation, stimulation of the renin-angiotensin-aldosterone system may be crucial to maintain high blood pressure, which could explain the exquisite sensitivity to very low doses of inhibitors of the renin-angiotensin system (Hilgers et al., 2001). In accordance with this observation, we previously reported that the occurrence of malignant hypertension in the 2K1C rat model can be prevented by low-dose angiotensin II type 1 receptor blockade (Hilgers et al., 2001). A similar phenomenon has been described in another renin-driven transgenic model (Whitworth et al., 1995) of hypertension.

In some regards, malignant and non-malignant may appear as merely ends of a spectrum, but there was a rather clear-cut difference regarding capillary supply and neovascularization: There was simply no impairment in non-malignant hypertension but a marked decrease in malignant hypertensive rats. In fact, there was even a trend toward increased capillary supply in the contralateral kidney of non-malignant hypertensive rats which might be explained by some degree of compensatory hypertrophy (Schwartz et al., 1993). Previous findings of increased VEGF in hypertension (Belgore et al., 2001; Advani et al., 2007) are in line with our own observations of an increase in renal VEGF A expression in both malignant and non-malignant hypertension. Moreover, we detected an induction of several VEGF isoforms and receptors in the kidneys of hypertensive rats. In contrast to the impaired capillary supply, the expression of VEGFs and VEGF receptors were not reduced in malignant hypertension, with the exception of myocardial VEGF A. Some renal VEGF isoforms and plasma VEGF levels were even higher in malignant hypertension than in non-malignant hypertension and controls. Thus, capillary rarefaction in malignant hypertension cannot be explained as a consequence of reduced expression of these angiogenic factors. Rather, overexpression of VEGF as seen in various hypertensive models (Advani et al., 2007; Belabbas et al., 2008) might serve a protective function. However, overexpression of VEGF isoforms and VEGF receptors as seen in our animal model neither protected against the development of malignant hypertension nor against capillary rarefaction. The increased expression of angiopoietin-2 in the right kidney of malignant hypertension might be interesting and worthy of further study in view of previous reports on the association of circulating angiopoietin-2 with renal injury in chronic kidney disease (Chang et al., 2013; Tsai et al., 2014). However, this pattern of expression was limited to the right kidney, and was not present in the left ventricle. Such an observation thus appears unlikely to explain the capillary rarefaction which was seen in both organs.

Malignant hypertensive rats exhibited a more pronounced stimulation of plasma renin and aldosterone, as described previously (Möhring et al., 1976). In other models of hypertension, high aldosterone leads to an overexpression of PAI-1 (Brown et al., 2000; Ma et al., 2006). The effects of PAI-1 on vessel formation are quite complex (Stefansson et al., 2003), but the protein can certainly inhibit angiogenesis *in vivo* (Stefansson et al., 2001). We demonstrated that malignant hypertension, impaired disc neovascularization and reduced capillary supply of target organs are associated with elevated expression of PAI-1 in the heart and the contralateral kidney but our study is limited in that we cannot establish a cause-and-effect relationship. Further, we cannot exclude the possibility, that the more severe renal injury in malignant hypertension could in turn cause an impairment of angiogenic processes. Such an impaired post-ischemic angiogenesis has been described previously in a normotensive rat model of more severely impaired kidney function (5/6 nephrectomy) of longer duration (Jacobi et al., 2006).

While we are at present unable to clearly delineate a mechanism, our data show that only malignant hypertension, not non-malignant hypertension, is characterized by a reduced disc neovascularization and by a reduced capillarisation of the kidney and heart. This observation may lead to a better understanding of the puzzling relationship between vasculogenic processes, hypertension and target organ damage. Moreover, therapeutic interventions to induce a coordinated program stimulating angiogenesis (for example via Hypoxia-Inducing Factors) may hold promise to prevent progressive target organ damage in malignant hypertension.

## Author contributions

AH acquired data, interpreted the results, and drafted the manuscript. LJ and NC acquired data, interpreted the results, and revised the manuscript. JJ and KH designed the work, interpreted the data, and drafted the manuscript. RV and KA designed the work and revised the manuscript. All authors gave final approval of the manuscript to be published.

## Funding

This study was supported by a grant from the Interdisciplinary Center for Clinical Research (IZKF) at the University Hospital of the University of Erlangen-Nuremberg to KA and KH (project F1) and by a grant from the Doktor Robert Pfleger-Stiftung to AH and KH.

### Conflict of interest statement

The authors declare that the research was conducted in the absence of any commercial or financial relationships that could be construed as a potential conflict of interest.
