# Supplementary material for: Impaired Neovascularization and Reduced Capillary Supply in the Malignant vs. Non-malignant Course of Experimental Renovascular Hypertension
Source: Front Physiol. 2016 Aug 30;7:370. doi: 10.3389/fphys.2016.00370 (PMC5003830; doi:10.3389/fphys.2016.00370)
Supplement: Supplementary file 1 [file Table1.PDF]

Table S1: Body and organ weights, physiologic parameters und markers of organ damage in all experimental groups including the undetermined animals.

	**Sham-OP control**	**Non-malignant hypertension**	**Malignant hypertension**	**Undetermined hypertension**
Body weight at OP (g)	151.2±1.4	151.7±1.8	151.8±2.4	157.1±2.5
Body weight 18 days after OP (g)	292.9±4.1	279.2±6.3	240.3±10.1 * †	255.0±8.0 *
Body weight at sacrifice (g)	381.0±8.0	343.6±9.4 *	251.4±7.5 * †	315.5±11.2 * ‡
Kidney weight (right, unclipped) (g)	1.22±0.02	1.44±0.06 *	1.40±0.10	1.73±0.07 * # ‡
Kidney weight (left, clipped) (g)	1.20±0.03	0.94±0.07 *	0.86±0.08 *	1.14±0.04 ‡
Left ventricular weight (g)	0.78±0.01	0.93±0.09	0.85±0.08	1.00±0.09
Relative left ventricular weight (mg/g)	2.05±0.04	2.69±0.26	3.42±0.31 *	3.18±0.26 *
Heart rate (bpm)	461.2±13.4	441.0±11.2	467.1±21.4	435.2±29.1
Serum creatinine (mg/dl)	0.20±0.01	0.22±0.01	0.47±0.06 * †	0.29±0.03 ‡
Serum urea (mg/dl)	37.7±1.1	45.0±2.9	122.3±18.3 * †	75.5±10.2 * ‡
Renin in clipped kidney (juxtaglomerular index, %)	22.4±1.5	30.5±1.8 *	31.2±3.0 *	33.6±2.0 *
Serum aldosterone (pg/ml)	329±32	2007±732	3927±942 *	2690±737 *
Collagen I deposition in unclipped kidney (% area stained)	4.66±0.44	4.98±1.02	10.16±1.53 * †	7.89±1.24
Capillaries in the kidney (number per cortical view)	29.2±1.2	33.5±1.3	24.1±0.8	29.4±3.7
Capillaries in the left ventricle (number per view)	199.4±5.3	183.8±3.5	141±8.1 * †	173.3±7.5 * ‡
Disc angiogenesis (% area)	81±3	80±2	67±3 * †	73±3

Data are means ± sem. * $p<0.05$ versus sham, † $p<0.05$ versus non-malignant hypertension, ‡ $p<0.05$ versus malignant hypertension.
