# Supplementary material for: Impaired Neovascularization and Reduced Capillary Supply in the Malignant vs. Non-malignant Course of Experimental Renovascular Hypertension
Source: Front Physiol. 2016 Aug 30;7:370. doi: 10.3389/fphys.2016.00370 (PMC5003830; doi:10.3389/fphys.2016.00370)
Supplement: Supplementary file 2 [file Image1.PDF]

Figure S2: Systolic blood pressure (mm Hg) as measured weekly by tail cuff plethysmography during the experimental time from day 3 before clipping until day 32 after clipping of the left renal artery.

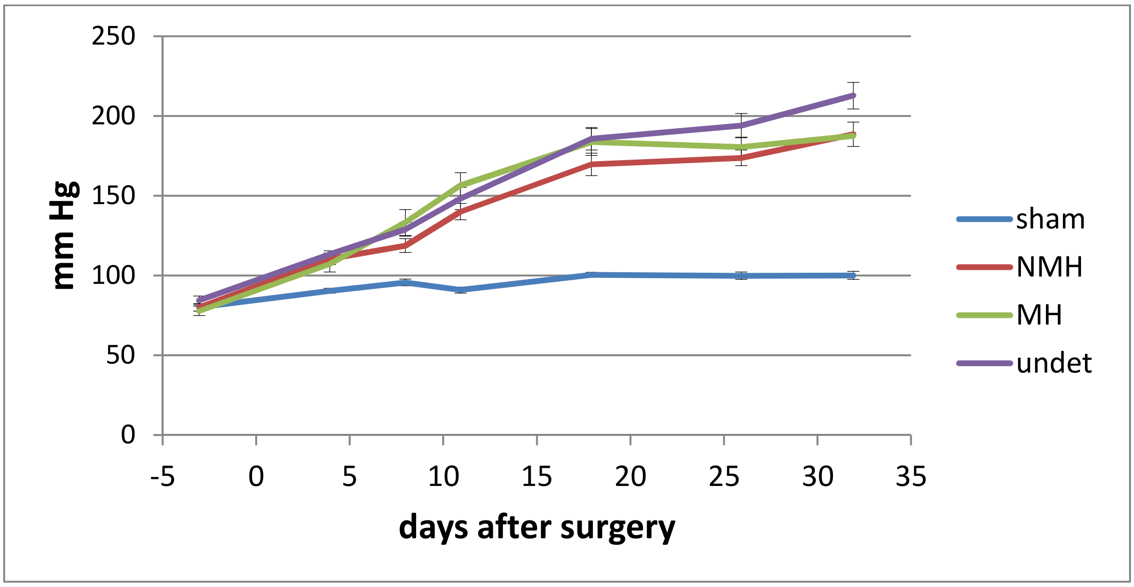

Shown are measurements of all experimental groups including the undetermined animals. Sham=sham operated controls, NMH=non-malignant hypertension, MH=malignant hypertension, undet=undetermined group.
