# Supplementary material for: Impaired Neovascularization and Reduced Capillary Supply in the Malignant vs. Non-malignant Course of Experimental Renovascular Hypertension
Source: Front Physiol. 2016 Aug 30;7:370. doi: 10.3389/fphys.2016.00370 (PMC5003830; doi:10.3389/fphys.2016.00370)
Supplement: Supplementary file 3 [file Image2.PDF]

Figure S3: Body weight (g) as measured daily throughout the duration of the experiment.

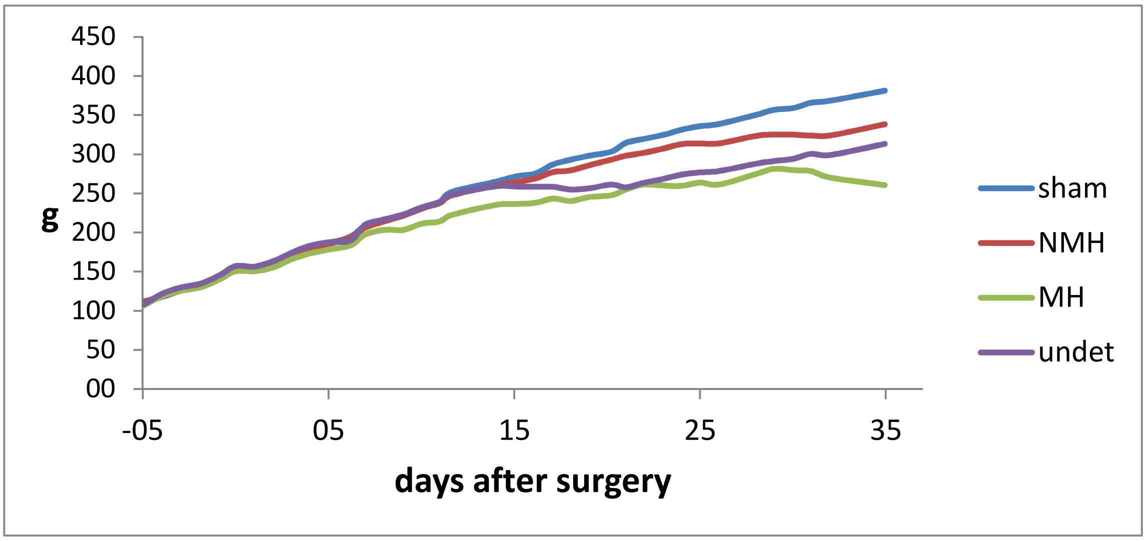

Shown are measurements of all experimental groups including the undetermined animals. Sham=sham operated controls, NMH=non-malignant hypertension, MH=malignant hypertension, undet=undetermined group.
