# Supplementary material for: Impaired Neovascularization and Reduced Capillary Supply in the Malignant vs. Non-malignant Course of Experimental Renovascular Hypertension
Source: Front Physiol. 2016 Aug 30;7:370. doi: 10.3389/fphys.2016.00370 (PMC5003830; doi:10.3389/fphys.2016.00370)
Supplement: Supplementary file 4 [file Image3.PDF]

Figure S4: Plot showing body weight gain against renal vascular lesion number of rats with malignant and non-malignant hypertension.

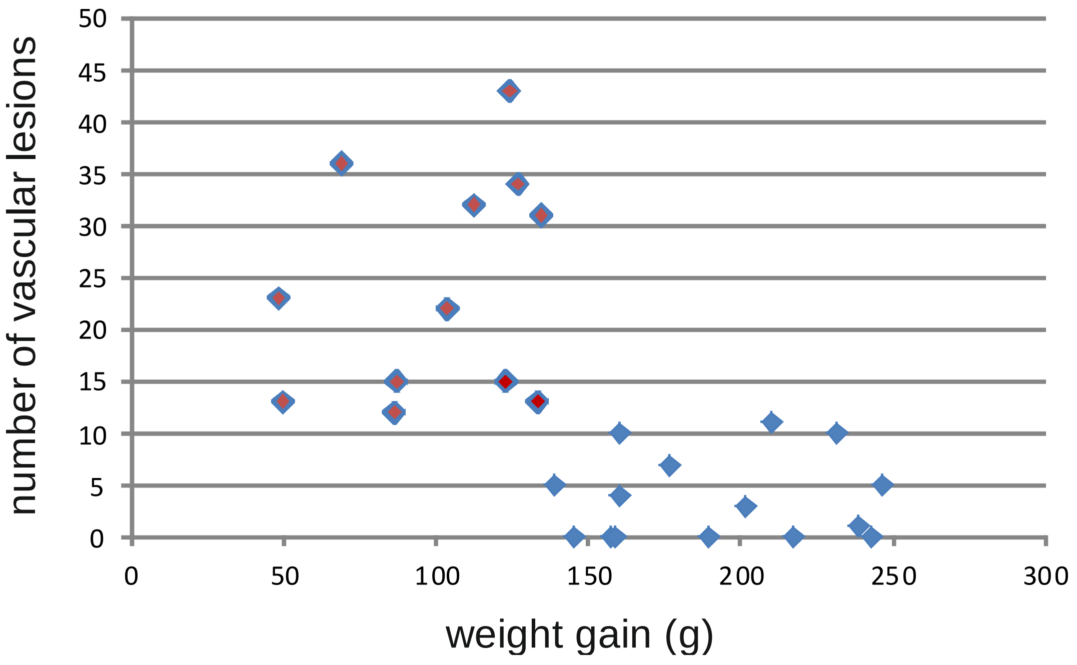

The occurrence of malignant hypertension was defined as the presence of weight loss in these otherwise still growing rats and characteristic vascular lesions in the contralateral kidney exposed to high blood pressure. Therefore we performed split-half analyses for both criteria: weight loss and number of characteristic vascular lesions (onion skin lesions and fibrinoid necroses). To avoid the need for arbitrary definitions of “weight loss”, the actual weight gain between the 2K1C procedure and the end of the study was used in the split-half analysis for “weight loss”. In these growing rats, the animals with the most weight loss had obviously the lowest weight gain during this time. Hypertension was considered malignant if a rat was in the upper 50% for both criteria (n=13, MH), or non-malignant if animal was lower 50% for both criteria (n=15, NMH). Red symbol, malignant hypertensive rats; blue symbol, non-malignant hypertensive rats.
