# Supplementary material for: Impaired Neovascularization and Reduced Capillary Supply in the Malignant vs. Non-malignant Course of Experimental Renovascular Hypertension
Source: Front Physiol. 2016 Aug 30;7:370. doi: 10.3389/fphys.2016.00370 (PMC5003830; doi:10.3389/fphys.2016.00370)
Supplement: Supplementary file 5 [file Image4.PDF]

Figure S5: Renin, collagen and PCNA stainings in renal tissue

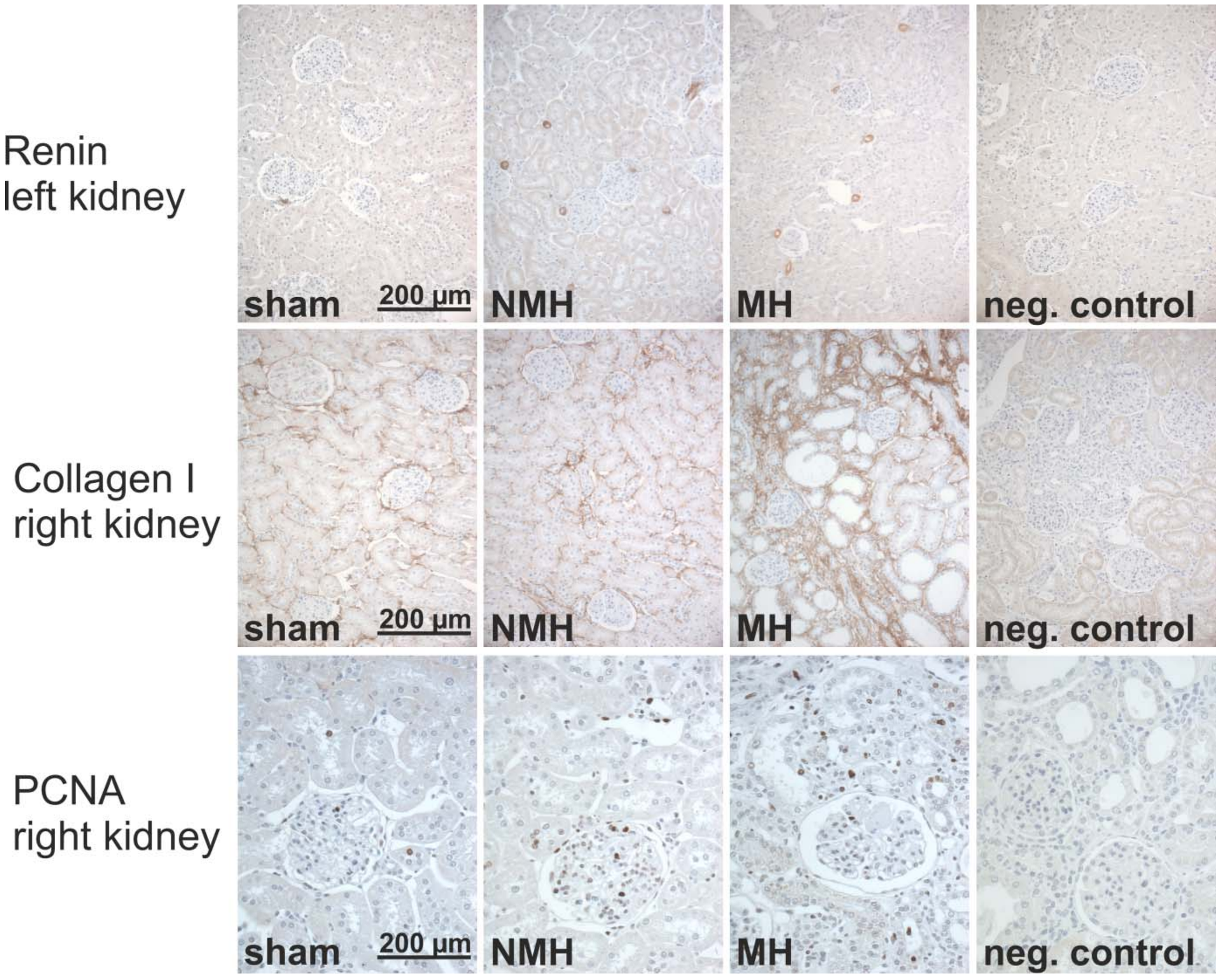

Representative photomicrographs of renin, collagen I and PCNA staining in the kidneys of experimental groups. Sham=sham operated controls, NMH=non-malignant hypertension, MH=malignant hypertension, neg. control=IgG control staining for specificity of antibody.
